# Sickness presenteeism explained by balancing perceived positive and negative effects

**DOI:** 10.3389/fpsyg.2022.963560

**Published:** 2022-08-25

**Authors:** Daniela Lohaus, Wolfgang Habermann, Malte Nachreiner

**Affiliations:** ^1^Business Psychology Institute, Social Sciences Faculty, Darmstadt University of Applied Sciences, Darmstadt, Germany; ^2^H&L Karriereberatung, Lautertal, Germany

**Keywords:** presenteeism, absenteeism, behavioral consequences, health belief model, expectancy theory

## Abstract

Within the ever-growing body of research on sickness presenteeism, studies of perceived consequences are scarce and equally rare are joint considerations of beneficial and harmful effects. This study examined how experienced and expected consequences of the behavior are related to presenteeism. Positive and negative effects were considered simultaneously and comprehensively. This approach allowed us to capture the trade-off process of individuals in deciding to work or call in sick when ill. In a cross-sectional online survey, 591 working adults in Germany rated a thoroughly developed pool of specific experienced or potential consequences of working while sick and gave an overall judgment of effects. The results show that perceptions of effects are consistent with behavior. Individuals who exhibit presenteeism do so primarily because of work-related effects such as the completion of one’s work tasks and the meeting of deadlines. Few specific effects stand out and can largely explain attendance behavior and the overall assessment of effects. The findings are consistent with the assumptions of the health belief model and the expectancy value theory of work motivation and they relate to the health-performance framework. They demonstrated that benefits and costs of the behavior are simultaneously weighed in the decision to engage in presenteeism or not.

## Introduction

Sickness lies on the continuum between complete health without any complaints and severe manifest illness based on objective medical criteria that require professional medical treatment (Steinke and Badura, 2011; Ruhle et al., 2020). In terms of work ability, the space between the poles represents the “gray zone” of relative illness that provides a factual but not a compelling reason for sick leave (Steinke and Badura, 2011), i.e., it does not go along with a certified inability to work. In this gray area, usually workers decide on the basis of their subjective perception whether to work (sickness presenteeism) or to call in sick (sickness absenteeism; Gerich, 2015; Johnson et al., 2018).

In this decision between presenteeism and absenteeism, affected individuals consider the expected consequences with their probability of occurrence and their significance for themselves (Lohaus and Habermann, 2021). These assessments are based on their experience and past behavior (Ajzen, 1991) as well as on expectations and beliefs (Vroom, 1964, 1995), such as what consequences people expect for attending or not attending work when ill (Johansen et al., 2014; Cooper and Lu, 2016; Biron et al., 2021).

There is literature on both, experienced and presumed consequences of presenteeism. The predominant view is that presenteeism has primarily negative consequences, especially for the long-term health of those affected, their work ability, and productivity (e.g., Collins et al., 2005; Gustafsson and Marklund, 2011; Taloyan et al., 2012; Conway et al., 2014; Miraglia and Johns, 2016; Skagen and Collins, 2016; Strömberg et al., 2017; Gosselin, 2018; Chen et al., 2021). Some researchers attempted to estimate the involved costs for employers (e.g., Nagata et al., 2018) and in some cases, the calculated productivity loss caused by presenteeism was assessed higher than the costs of absenteeism (e.g., Stewart et al., 2003). Such a comparison of figures might well be correct, but it overlooks that any case of presenteeism means at least some productivity in comparison to zero productivity due to absenteeism (Vingård et al., 2004; Johns, 2010; Arnold, 2016; Lohaus and Habermann, 2019). The productivity advantage of presenteeism over absenteeism may account for the fact that some organizations tolerate presenteeism (Thun et al., 2013; Aronsson and Marklund, 2018; Ferreira et al., 2019; Ruhle and Süß, 2020). Nevertheless, most publications advice practitioners to reduce presenteeism in organizations by actions of the management in dealing with this phenomenon (e.g., Cancelliere et al., 2011; Justesen et al., 2017; Komp et al., 2022).

It is only relatively recently that (potential) positive effects of presenteeism have been given more attention (e.g., Demerouti et al., 2009; Garrow, 2016; Giæver et al., 2016; Miraglia and Johns, 2018; Lohaus et al., 2021; Wang et al., 2022). However, these are mostly either conceptual considerations (e.g., Miraglia and Johns, 2018; Whysall et al., 2018; Karanika-Murray and Biron, 2020) or only a few (potential) effects are considered at a time (e.g., Giæver et al., 2016; Lohaus et al., 2021). Miraglia and Johns (2018) and Karanika-Murray and Biron (2020) argue that in certain situations, presenteeism might be beneficial for workers’ mental health and well-being and even have a therapeutic effect for the affected individuals while helping them to balance health impairments with performance requirements. Empirical studies support this assumption. For example, affected individuals report that presenteeism had the positive psychological effect for them of not letting the illness get them down (Lohaus et al., 2021). Further, presenteeism was found to be useful for obtaining better performance evaluations under conditions of heavy workload (Wang et al., 2022). Several studies examined the reasons for sickness presenteeism. Some of the reasons can be understood as experienced or anticipated effects of the behavior and thus might be an indirect measure of effects. Lu et al. (2013) and Gerich (2020) distinguished reasons for presenteeism according to their direction into approach and avoidance motives. Accordingly, presenteeism can be understood as the attempt to achieve positive consequences and to avoid negative ones. For example, Johansen et al. (2014) stated that individuals worked despite illness because it was positive for their health. Ruhle and Schmoll (2021) found that people opted for presenteeism to avoid the work piling up until their return. Having no replacement is a common argument among self-employed (Vinberg et al., 2021). In several studies, the most important reported reason for presenteeism was that people did not want to burden their colleagues (Al Nuhait et al., 2017; Bachert et al., 2017; Navarro et al., 2018).

To date, we have not identified any studies that directly examine an entirety or a comprehensive list of effects of presenteeism which employees consider when deciding to work in case of perceived illness or not. Such assessments would have to refer to relevant positive and negative (potential) effects simultaneously. This is important because these subjective evaluations guide behavior. Although both, positive and negative effects have been reported (e.g., Ruhle et al., 2020; Chou and Mach, 2021; Lohaus et al., 2021), it has not yet been demonstrated that in case of presenteeism perceived positive consequences outweigh perceived negative ones. Knowledge of according perceptions would offer insight as to possible starting points for managing presenteeism in organizations with regard to both, economic and health-related conditions (e.g., Ammendolia et al., 2016).

The current study has two objectives: First, we strive to show how differences in perception of the effects of presenteeism relate to differences in illness behavior. Second, we want to explore which single effects have the strongest relationship with the behavior of working individuals and the greatest impact on the overall assessment of effects. To achieve these goals, we investigate (potential) positive and negative effects of presenteeism simultaneously and thus merge research previously separated by the direction of effects.

## Research questions and hypotheses

Is has been demonstrated that affected employees base their decision to work despite illness or not on their goals and expectancies of consequences (Lohaus and Habermann, 2021) as postulated by the expectancy value theory of work motivation (VIE) by Vroom (1964, 1995, 2005). The health belief model—HBM—(Rosenstock, 1966; Rosenstock et al., 1988) is an expectancy-value theory commonly used for the study of health-related behavior (e.g., Janz and Becker, 1984; Bakker et al., 1997; Fall et al., 2018; Wong et al., 2020; Zampetakis and Melas, 2021), such as preventive and sick role behavior (Abraham and Sheeran, 2015). The HBM explains rational behavior under conditions of uncertainty and proposes that health-related behavior is a result of the individual’s evaluation process (Rosenstock et al., 1988). In accord with expectancy-value theory, the process incorporates the individual’s expectations of the consequences of one’s actions, the expectancies about one’s competence to exhibit the behavior required to influence the outcomes, and the subjective value of the outcomes. That is, individuals engage in a health-related behavior if they perceive their current lifestyle (e.g., smoking) as a threat to a valued outcome (e.g., physical and mental well-being), and if they are able to adopt a specific risk-reducing behavior (e.g., reducing daily nicotine consumption). Although different conceptualizations and operationalizations of the HBM exist, there is consensus regarding the core variables (Carpenter, 2010; Jones et al., 2014; Green et al., 2021). According to the theory, health-related behaviors are explained by individuals’ perceptions of four variables. These are on the one side, (1) benefits and (2) costs of or barriers to the behavior, which people weight against each other. On the other side, people consider health threats, which imply subjective perceptions of (3) one’s vulnerability and (4) the severity of health problems. According to meta-analytic results, perceived benefits and barriers are the strongest predictors for health-related behavior (Carpenter, 2010). Therefore, we will focus on them in the current study.

The HBM states that in order to increase the likelihood of a distinguished health-related behavior (such as being vaccinated or ceasing to smoke), the individual must perceive strong positive effects of this behavior. The meta-analyses by Harrison et al. (1992) and Carpenter (2010) confirm this assumption. They report correlations of 0.13 and 0.27 between perceived benefits and behavior. Hence, we expect a positive correlation of anticipated or experienced consequences with presenteeism behavior in case of employees feeling sick:

*Hypothesis 1*: The more positive the perceived effects of presenteeism, the higher is the propensity to show presenteeism

As with many health-related behaviors (such as being vaccinated or not), the decision between working despite illness and calling in sick is dichotomous, as it only refers to whether individuals choose to work or not, not how much they work (Gerich, 2015; Lohaus and Habermann, 2021; Rivkin et al., 2022; Whysall et al., in press). In practice, the dichotomous decision often arises for legal reasons. Employees are required to report sick before the start of their working hours, stating the expected duration (Continuation of Remuneration Act §5). The relevant paragraph of the law refers to days, not hours worked. Further, Rivkin et al. (2022) and Whysall et al. (in press) argue that individuals decide about presenteeism on a daily basis according to the expected gain from presenteeism with respect to fulfilling their performance requirements. The fact that some employees opt for presenteeism at least in some cases of sickness while others decide for absenteeism is assumed to be associated with differences in perceptions of consequences of the respective behavioral alternatives. Therefore, we hypothesize:

*Hypothesis 2*: Individuals who exhibited presenteeism perceive the effects significantly differently from those who did not exhibit presenteeism

Further, relating to the HBM and the VIE-model, one could assume that people who opted for presenteeism in the past see the overall effects of presenteeism in a positive light, i.e., positive assessments outweigh negative ones, because otherwise they might not have decided for presenteeism. However, if they exhibited presenteeism and experienced negative consequences, they might be expected to show a lesser degree of presenteeism in the future. This implication is captured in Hypothesis 1. In contrast, employees who decided against presenteeism in the past probably did so, because overall they evaluated the effects of presenteeism as negative. Thus, we hypothesize that the quality of the evaluation of effects is consistent with the individuals’ past behavior:

*Hypothesis 3a*: Overall, individuals who chose presenteeism in case of sickness perceive its effects positively
*Hypothesis 3b*: Overall, individuals who did not choose presenteeism in case of sickness perceive its effects negatively

Regarding the understanding of presenteeism as well as its management in organizations, a further question is relevant. As there is an abundance of (potential) positive and negative effects of presenteeism, it is unlikely that each effect contributes with the same weight to the eventual decision. To date, we have no reliable knowledge of the relative weights of effects. Therefore, it would be helpful to identify those with the greatest impact on the behavior of employees. This knowledge would allow researchers to construct more expedient and less tiresome questionnaires and so gain easier access to the underlying relevant effects. Organizational practitioners would be able to focus their interventions on the effects with the greatest chances of success. This leads to the exploratory research question:

*Research question 1*: Which perceived effects best predict presenteeism?

Past research has suggested that the decision for or against presenteeism might depend on the attitudes of the individuals concerned toward this behavior (Johansen et al., 2014; Krane et al., 2014; Miraglia and Johns, 2016; Rebmann et al., 2016; Lohaus and Habermann, 2019). With respect to work-related attitudes, a distinction is frequently made between general attitudes and specific facets (Judge and Kammeyer-Mueller, 2012). Researchers assume a hierarchical structure of attitudes in the way that the general or overall attitude represents a general factor. Accordingly, overall assessments, which often consist of only one item, are used instead of or alongside more differentiated instruments to measure the specific facets on a continuum of positive to negative evaluations (Judge et al., 2017). Therefore, it is plausible to make this distinction for presenteeism. When employees wake up in the morning and realize that they are sick, it is conceivable that they make their decision on the basis of a general assessment of whether it is favorable or unfavorable to work despite illness. It is equally conceivable that they weigh different (potential) outcomes. Thus, for this research, we will use a composite or overall single-item assessment of effects to record participants’ evaluation of the effects of presenteeism as a whole (Judge et al., 2017) as well as a differentiated measure of various specific effects. This procedure will allow the investigation of how the evaluation of single effects relates to the overall judgment and which specific effects have the greatest impact on this judgment. This leads to the following research question:

*Research question 2*: How well does the evaluation of the single effects explain the overall assessment of effects and which effects best explain the overall assessment?

Conceptual work and research on presenteeism have revealed that some factors that strongly influence presenteeism behavior can be interpreted according to the HBM as barriers or facilitating factors, depending on their direction of influence. The individuals’ health (representing the vulnerability and the severity in terms of the HBM) is one of the most important person-related factors influencing presenteeism (Miraglia and Johns, 2016; Hirsch et al., 2017). With regard to job demands, workload has turned out to be a very strong impact factor in the sense that workload is positively associated with presenteeism (e.g., Demerouti et al., 2009; Miraglia and Johns, 2016; Pohling et al., 2016; Baeriswyl et al., 2017; Brborovic and Brborovic, 2017; Saijo et al., 2017; Wang et al., 2022). On the other hand, job control or adjustment latitude understood as workers’ options to reduce their work output or adapt work practices in response to discomfort (Johansson and Lundberg, 2004) has been identified as an important personal resource that reduces presenteeism (e.g., Gerich, 2014; Jourdain and Vézina, 2014; Oshio et al., 2017; Mach et al., 2018). As these factors might influence the perceived effects of presenteeism independently of the individual’s attitudes, they served as control variables in the current study.

## Materials and methods

### Procedure

The study design was a cross-sectional online survey conducted during the COVID-19 pandemic in Germany. We contracted a commercial panel provider for digital studies to invite working adults to the study in order to reach a broad range of employees (Aguinis and Bradley, 2014; Landers and Behrend, 2015; Walter et al., 2019). Participants received a small monetary reward for their participation. After the participants provided their written informed consent to participate, the survey started. The survey was online from 04.02.2022 until 09.02.2022. Of 836 people who accessed the survey home page, 55 did not start and 658 completed the survey (78.7%).

### Questionnaire

To create a comprehensive pool of possible effects of presenteeism, we drew on the systematically derived collection of items that had already been created for a previous study (for details, see Lohaus and Habermann, 2021). This pool covered all relevant areas recently identified in a systematic review on presenteeism; i.e., the individual, the work, the organization, and the environment (Lohaus and Habermann, 2019). To keep the size of the questionnaire as small as possible, the original pool of 58 items was reduced to a total of 45 effects of presenteeism by deleting or combining similar phrases (see lower part of Table 1). These effects formed the main part of the survey.

**Table 1:** Mean and SDs for single-item overall assessment, mean across 45 effects, 45 single effects for those who were ill and exhibited presenteeism (P +) and those who were ill and did not exhibit presenteeism (P −).

	P + (*N* = 326)^**^		P − (*N* = 123)^**^	
	*M* ^*^	*SD*	*M* ^*^	*SD*
Single-item overall assessment	−9.72	42.33	−43.15	46.37
Mean across 45 effects	6.86	39.48	−14.60	44.77
The completion of my work tasks and meeting deadlines	24.68	40.49	−4.65	47.87
The security of my job	23.87	40.11	−0.99	42.54
My standing with my manager	22.67	35.16	1.51	43.44
My perception by others as reliable and professional	21.94	39.02	−13.74	47.33
The achievement of the company’s economic goals	21.16	39.97	−1.87	44.72
My standing in the organization	20.76	32.34	−2.73	42.31
My reputation among my colleagues	20.71	37.07	−7.88	46.20
The evaluation of my performance by my supervisor	20.16	37.06	−5.50	45.03
The workload for my teammates	18.25	38.35	−3.77	45.22
My professional skills	17.51	33.48	−10.33	44.33
The care for and satisfaction of my contacts (customers. Patients. etc.)	17.31	38.92	−3.34	43.89
My income	15.58	35.67	10.17	35.29
My professional development opportunities	15.27	37.70	−3.98	38.16
My employability	15.10	38.07	−0.61	41.22
The social insurance systems	14.49	36.85	0.67	40.60
My involvement in important tasks and decisions	14.30	42.30	−13.25	48.37
My future freedoms in the completion of my tasks	13.75	38.51	−9.37	46.13
The reputation of my organization in the public	11.34	30.77	−6.32	41.03
My colleagues’ work ethic	10.92	36.63	−6.46	41.20
The mood/atmosphere in the team	10.89	36.03	−8.83	41.69
The support from my manager	10.57	41.35	−8.34	43.66
My dedication and work ethic	10.40	49.90	−14.75	51.65
The way the organization treats me (e.g. regarding fairness and equal treatment)	9.46	42.59	−9.41	45.87
The prosperity of our society	8.96	35.04	−1.63	35.84
HR-policy measures (e.g. interview because of sick leave, reintegration)	8.74	33.67	−10.29	43.16
Maintaining my social contacts at work	8.18	39.25	−9.22	44.11
My future workload	6.99	39.46	−13.76	47.91
My relationship with my family and friends	6.46	38.13	−15.68	49.28
The economic situation of our country	6.29	36.99	−5.67	43.59
My creativity and acceptance of innovations	6.23	35.07	−15.60	46.18
The distraction from my symptoms of illness	4.50	40.91	−14.61	53.04
My usual daily routine	4.41	42.32	−15.80	48.82
The quality of my work/my performance	3.20	42.88	−30.13	48.97
The values of our society	2.55	37.14	−13.38	38.93
The health of my teammates	−1.16	40.54	−32.81	50.68
My future efficiency	−1.40	43.12	−28.98	45.64
The completion of private tasks on the way to and from work	−6.62	43.76	−24.11	47.78
The joy I get from my work	−7.10	47.29	−27.79	47.65
Occupational safety (e.g. prevention of errors and accidents at work)	−7.24	40.51	−39.33	48.62
My future susceptibility to disease	−7.70	39.18	−31.11	46.22
My mental health	−16.32	46.50	−37.75	45.38
My work-life balance	−17.85	44.46	−35.59	43.20
My leisure activities	−19.54	41.03	−41.29	42.50
My stress	−26.77	44.49	−39.25	45.33
My physical health	−26.99	46.59	−49.28	44.21

* Effects were rated on a slider ranging from − 100 to + 100;

** P+: Participants who reported presenteeism days, i.e., had a presenteeism propensity of > 0.0; P−: Participants who reported no presenteeism days, i.e., had a presenteeism propensity of 0.0.

The first question in the survey was a filter question to ensure that participants were employed in the past year. Participants were asked what percentage of their work time they had spent in the past year working from home and at workplaces within the organization. Presenteeism and absenteeism were then covered with one question each: “Please think now about the past 12 months and your state of health on working days. On how many working days did you (regardless of the place of work) … work even though you were sick?… called in sick and did not work?” (e.g., Ruhle et al., 2020). Depending on whether the participants had indicated presenteeism days or not, they were asked to think of the days in the past 12 months on which they had worked despite illness or to imagine that they had worked despite illness. Next, they should assess the impact of this behavior on the aspects listed on the following pages, while considering simultaneously the importance and the strength of the impact. All of the 45 above-mentioned effects were to be assessed, presented in randomized order (*α* = 0.98). In addition, participants gave a single-item overall assessment of effects of presenteeism either before or after the presentation of the 45 specific effects. All effects were scored on a slider with endpoints marked “negative” and “positive.” Not visible to the participants was the underlying scale from −100 to +100.

Three control variables were considered due to their potential impact on the decision for or against presenteeism. Job demands and specifically workload correlate highly with presenteeism (Miraglia and Johns, 2016) and adjustment latitude or job control is perceived as a positive resource, which might help the individual “to buffer negative health effects induced by work demands” (Gerich, 2019, p. 96). Further, poor health is strongly associated with presenteeism (Miraglia and Johns, 2016). Thus, individuals with high workloads, little control and poor health are more likely to opt for presenteeism in case of sickness. To control for these potential influences, subjects were asked to describe their work situation with regard to their subjectively perceived workload (Weyer et al., 2014) on three items (*α* = 0.82; e.g., “My workload is very high.”) and their adjustment latitude (Johansson and Lundberg, 2004; Gerich, 2019) on four items (*α* = 0.66; e.g. “Do you have the option to adjust your workload when you feel unwell or unbalanced?”). Further, they rated their health on four items with the following instruction: “Please rate your health based on the following statements between the two endpoints mentioned:” (Lohaus and Röser, 2019; *α* = 0.80). An example item is “My current state of health is…” followed by the endpoints “very bad” and “very good.” All control variables were rated on five-point scales. The last part of the questionnaire concerned demographic variables. Three items were included to assess the attention of participants and seriousness of participation.

We calculated the sickness presenteeism prevalence as the percentage of participants having shown presenteeism (at least 1 day) during the 12 month before the survey. The number of health events (sick days) was determined as the sum of the presenteeism and absenteeism days (Gerich, 2016; Lohaus and Röser, 2019). We computed presenteeism propensity as presenteeism days divided by the number of sick days (Biron et al., 2006; Gerich, 2014, 2016; Lohaus and Röser, 2019). We used presenteeism propensity as the indicator of presenteeism behavior. It reflects an individual’s probability of opting for sickness presence rather than sickness absence in case of illness (Gerich, 2016) and thus reflects the decision of the individuals rather than their vulnerability (Gerich, 2015).

### Data processing

First, we eliminated the data of individuals who answered more than one of the three attention control questions incorrectly. Thus, 591 participants remained in the data set. Data analysis was performed using IBM SPSS version 27.0 (IBM SPSS Inc., Chicago, Illinois). The level of statistical significance was set at *p* < 0.05 in all the analyses conducted.

Frequencies, ranges, means, and SDs were determined to describe the sample. Since we investigated how perceived effects relate to health-related behavior, we included only those participants in the analyses that were ill during the previous year (sick days >0) because only those had to choose between presenteeism and absenteeism. That resulted in a sample size of *N* = 449. We used two indicators of perceived effects of presenteeism: the overall assessment of effects (single item) and the calculated mean across the evaluation of 45 single effects.

To test the hypotheses and to investigate the research questions, we used Pearson’s correlation coefficient, *t*-test, Welch-Test, binary logistic regression analysis, and multiple linear regressions. For the investigation of the first research question, we formed extreme groups of presenteeism propensity; i.e. one group with a propensity of zero and the other with a propensity of >0.5. Thus, we compared those who never chose presenteeism with those who exhibited presenteeism for more than 50% of their sick days. This limit was chosen because participants with a lower propensity (0.5 or less) might be more similar to those who never chose presenteeism. This approach is also beneficial to equalize the size of the two samples (Rosnow and Rosenthal, 2003). Because several researchers suggest a ratio of 10 cases per variable (Moons et al., 2014; Pavlou et al., 2015), we applied forward and backward inclusion of variables to reduce the high number of variables (45 effects). Then, we matched participants by presenteeism propensity and repeated the procedure with the split samples. For the final model, we identified those variables that were significant predictors in the analyses of the complete sample and at least one of the split samples. To investigate research question 2, all participants who reported at least 1 day of sickness were included. Based on outlier analysis, data from four individuals were excluded (Tabachnick and Fidell, 2018). One item was eliminated due to intercorrelations of *r* > 0.80 with other items (Schroeder, 1990; Field, 2018), leaving 44 effects of presenteeism for the analysis.

## Results

### Sample

After the described data cleaning, 591 participants remained in the data set (34.3% female, 33.6% in a supervisory position). On average, the participants were 44.2 years old (*SD* = 11.7) and had a working experience of 23.3 years (*SD* = 12.7). More than 90% worked full time. The majority of them were employed (83.3%), 9.1% civil servants, and 5.1% self-employed. With regard to the highest educational qualification, 32% had completed vocational training and more than one-third held a university degree. Twenty-three percent worked in the service sector (public and personal services; public supply and disposal), around 16% in the industry, 11% in finance, IT, and corporate services, and 11% in trade, hospitality, and tourism. The rest worked in various sectors. A detailed description of the sample and the subsamples is presented in Table 2. It shows that the subsamples are relatively similar with regard to the demographic variables.

**Table 2:** Results of the descriptive analyses of demographic and employment-related characteristics of the study group.

	Complete sample *M* ± *SD* (Range)	Participants who reported sickness days *M* ± *SD* (Range)	Participants who reported presenteeism days (P+) *M* ± *SD* (Range)	Participants who reported no presenteeism days (P-) *M* ± *SD* (Range)	Participants with a presenteeism propensity > 0.5 (P++) *M* ± *SD* (Range)
*N*	591	449	326	123	187
Age (years)	44.2 ± 11.7 (19–69)	43.2 ± 11.8 (19–65)	42.2 ± 11.8 (19–65)	45.7 ± 11.2 (24–64)	42.5 ± 11.8 (19–64)
Gender (% female/male)^*^	34.3/65.7	38.3/61.7	40.5/59.5	32.5/67.5	39.6/60.4
Academics (%)	26.4	25.4	27.0	21.1	28.9
Employed (%)	83.1	85.5	85.0	87.0	83.4
Full time work (%)	91.2	91.5	91.7	91.1	92.0
Supervisory position (%)	33.6	33.9	36.3	27.5	38.0
Work experience (years)	23.3 ± 12.7 (1–50)	22.4 ± 12.6 (1–48)	21.4 ± 12.6 (1–47)	25.1 ± 12.2 (3–48)	21.6 ± 12.4 (1–47)
Work hours per week^**^	39.4 ± 7.3 (0–80)	39.6 ± 6.8 (0–80)	40.2 ± 6.4 (0–80)	38 ± 7.6 (0–60)	40.6 ± 7.2 (0–60)
Work place in organization (%)	76.4 ± 35.5 (0–100)	77.2 ± 35.1 (0–100)	75.6 ± 35.9 (0–100)	81.5 ± 32.5 (0–100)	70.2 ± 39.4 (0–100)
Home Office (%)	23.6 ± 35.4 (0–100)	22.8 ± 35.1 (0–100)	24.4 ± 35.9 (0–100)	18.5 ± 32.5 (0–100)	29.8 ± 39.4 (0–100)
Survey processing time (min.)	10.3 ± 8.1 (2.6–116.4)	10.6 ± 8.6 (2.8–116.4)	10.9 ± 9.5 (2.8–116.4)	9.7 ± 5.7 (3.6–36.0)	11.5 ± 11.3 (2.8–116.4)

* “non-binary” was an answer option, which was not selected by anyone.

** Participants reported their current working hours. These may be zero due to short-time work or unemployment as a result of the Corona pandemic or recent retirement.

### Descriptive results for subsamples

Table 3 lists the descriptive results for the study variables for the complete sample and the different subsamples used for the subsequent hypotheses testing and the investigation of the research questions. Obviously, the average amount of sick days is smaller for the complete sample than for the subsamples as the first column includes those participants who stated no sickness during the previous year. Column 4 presents the data of those who have been ill but never have chosen presenteeism. Thus, their prevalence and propensity necessarily is zero.

**Table 3:** Results of the descriptive analyses of work-related characteristics of the study group.

	Complete sample *M* ± *SD* (Range)	Participants who reported sickness days *M* ± *SD* (Range)	Participants who reported presenteeism days (P+) *M* ± *SD* (Range)	Participants who reported no presenteeism days (P-) *M* ± *SD* (Range)	Participants with a presenteeism propensity >. 5 (P++) *M* ± *SD* (Range)
Sick days	19.1 ± 34.9 (0–59)	25.1 ± 38.1 (1–250)	25.1 ± 35.6 (1–220)	25.3 ± 44.1 (1–250)	21.7 ± 38.0 (1–220)
Presenteeism (days)	7.7 ± 21.4 (0–220)	10.1 ± 24.1 (0–220)	14.0 ± 27.3 (1–220)	0	18.0 ± 34.4 (1–220)
Absenteeism (days)	11.4 ± 26.1 (0–250)	15.0 ± 29.0 (0–250)	11.1 ± 19.3 (0–150)	25.3 ± 44.1 (1–250)	3.6 ± 7.6 (0–60)
Presenteeism prevalence (%)	55.2	72.6	100	0	100
Presenteeism propensity	*	0.46 ± 0.39 (0–1)	0.64 ± 0.31 (0.02–1)	0	0.87 ± 0.16 (0.53–1)
Subjective health^**^	0.6 ± 0.9 (−2–2)	0.5 ± 0.9 (−2–2)	0.5 ± 0.9 (−2–2)	0.7 ± 0.9 (−1.67–2)	0.5 ± 0.9 (−2–2)
Workload^***^	1.9 ± 0.9 (0–4)	2.0 ± 0.9 (0–4)	2.1 ± 0.9 (0–4)	1.7 ± 0.9 (0–4)	2.1 ± 0.9 (0–4)
Adjustment latitude^**^	2.1 ± 0.8 (0–4)	2.0 ± 0.8 (0–4)	1.9 ± 0.8 (0.25–3.75)	2.3 ± 0.7 (0–4)	1.9 ± 0.8 (0.25–3.75)

* Presenteeism propensity can only be calculated for participants who reported sickness days.

** Five-point scale with −2 = “very bad/very low” to + 2 = “very good/very high.”

*** Five-point scale with 0 = “never” to 4 = “always.”

### Hypotheses testing

Hypothesis 1 proposed that a more positive perception of effects of presenteeism would be associated with a higher propensity to show presenteeism for those who reported sickness days. Thus, we expected a positive correlation for both the single-item overall assessment and the mean of all 45 effects with presenteeism propensity. Statistical analysis confirmed Hypothesis 1. The correlations between presenteeism propensity and the single-item overall assessment (*r_xy_* = 0.30, *p* < 0.001) and between presenteeism propensity and the mean of all 45 effects (*r_xy_* = 0.31, *p* < 0.001) were significant; representing medium sized relationships (Cohen, 1988). The control of possible influence variables (workload, adjustment latitude, and subjective health) by a partial correlation revealed the same pattern for the single-item overall assessment and the mean of effects (*r_xy_* = 0.34, *p* < 0.001 each), which means that these variables did not account for the significant relationship between presenteeism propensity and perception of favorability of effects.

Results also supported Hypothesis 2, which stated that individuals who exhibited presenteeism perceive the effects of the behavior significantly differently from those who did not exhibit presenteeism. On average, the single-item overall assessment of the group that had shown presenteeism (P+: *M* = − 9.72) differed significantly [*t*_(447)_ = − 7.27, *p* < 0.001] from that of the group that had not shown presenteeism (P-: *M* = − 43.15). The same pattern was found for the mean across all 45 items [P+: *M* = 6.86; P-: *M* = − 14.6; *t*_(187)_ = − 6.66, *p* < 0.001]. In both comparisons, Cohen’s *d* was 0.77, representing almost large effects. Results are displayed in the upper part of Table 1.

According to Hypothesis 3a, we expected that individuals who exhibited presenteeism perceive its effects positively; while individuals who did not choose presenteeism in case of sickness perceive its effects negatively (Hypothesis 3b). To test the hypotheses, we checked whether the mean evaluations and the single-item score differed significantly from zero in the expected direction. For those who reported presenteeism (Hypothesis 3a), both tests were significant. However only the mean across all 45 effects [P+: *M* = 6.86; *t*_(325)_ = 4.75, *p* < 0.001, Cohen’s *d* = 0.26] pointed in the expected positive direction. The difference for the single-item overall assessment [P+: *M* = − 9.72, *t*_(325)_ = −4.15, *p* < 0.001, Cohen’s *d* = 0.23] was negative and thus not in line with our expectations. Both effect sizes are small. Thus, Hypothesis 3a was only partly supported. We performed a *post hoc* analysis to check whether the above result could be due to the positioning of the single-item overall assessment of effects. The single-item rating taken at the beginning of the survey, i.e., before rating the 45 effects, (*M_b_* = − 19) versus after (*M_a_* = − 1.2) revealed a significant difference with regard to the positioning [*t*_(324)_ = − 3.87, *p* < 0.001, Cohen’s *d* = 0.43]. The evaluation at the beginning was more negative than at the end, both had a negative sign. This means, the negative perception of effects captured by the single item is not dependent on its position in the questionnaire. With regard to Hypothesis 3b, we found that participants who did not show presenteeism rated the single item significantly negative [P-: *M* = −43.15; *t*_(122)_ = −10.32, *p* < 0.001, Cohen’s *d* = 0.93] as well as the mean across all effects [P-: *M* = − 14.6; *t*_(122)_ = − 5.06, *p* < 0.001, Cohen’s *d* = 0.46]. These results were consistent with Hypothesis 3b and represented a large effect in the former case and a nearly medium sized effect in the latter.

### Investigation of research questions

Research question 1 that asked what perceived effects explained presenteeism propensity best was tackled by a binary logistic regression model that distinguished between participants who did not show presenteeism and those who had a high propensity (>0.5). The model was statistically significant, *χ^2^*_(5)_ = 84.8, *p* < 0.001, resulting in an acceptable to good amount of explained variance (Backhaus et al., 2018), as shown by Nagelkerke’s *R*^2^ = 0.32. Effect size calculated as Cohen’s *f^2^* was 0.48, which represents a strong effect (Cohen, 1988). Overall percentage of accuracy in classification (P++ vs. P-) was 76.5%, with a sensitivity of 59.3% and a specificity of 87.7%. Goodness-of-fit assessment indicated a good model. Table 4 presents the results for the complete subsample for those variables that were significant across the three analyses (one for the complete subsample and two for the split-half subsamples). Five effects significantly contributed to explain differences in presenteeism propensity. They all refer to the work context of the individuals and were “My perception by others as reliable and professional,” “The mood/atmosphere in the team”, “The health of my teammates”, “The completion of my work tasks and meeting deadlines”, and “My future efficiency.”

**Table 4:** Results of the binary logistic regression with degree of presenteeism propensity (high, zero) as dependent variable (significant variables only, *N* = 310).

	Participants who reported no presenteeism days (P-)		Participants with a presenteeism propensity >. 5 (P++)		*B*	*SE*	Wald	df	*p*	Exp(B)	95% CI for Exp(B)	
Variable	*M*	*SD*	*M*	*SD*							Lower bound	Upper bound
My perception by others as reliable and professional	−13.7	47.3	23.2	40.3	0.017	0.005	14.46	1	0.000	1.017	1.008	1.026
The mood/atmosphere in the team	−8.8	41.7	11.0	38.3	−0.017	0.005	9.88	1	0.002	0.983	0.972	0.994
The health of my teammates	−32.8	50.7	1.1	40.9	0.012	0.004	9.04	1	0.003	1.012	1.004	1.020
The completion of my work tasks and meeting deadlines	−4.7	47.9	29.3	41.5	0.011	0.004	7.96	1	0.005	1.011	1.003	1.018
My future efficiency	−29.0	45.6	4.9	43.8	0.009	0.004	5.25	1	0.022	1.009	1.001	1.016

Research question 2 investigated how well the evaluation of the specific effects explain the overall assessment of effects and which single effects best explain the overall assessment of experienced and envisioned effects. For this purpose, multiple linear regression analysis was used. The single effects statistically significantly explained the overall assessment of effects, *F*_(44,400)_ = 15.52, *p* < 0.001. Adjusted *R*^2^ for the overall model was 0.59, which indicates a high amount of explained variance according to Cohen (1988). Table 5 depicts the results for the complete sample of participants who reported sick days for those four variables that were significant. These were “My usual daily routine”, “My future efficiency”, “The values of our society”, and “The mood/atmosphere in the team.”

**Table 5:** Results of the multiple linear regression analysis with overall assessment of effects (single item) as dependent variable for the sample of participants who reported sick days (significant variables only, *N* = 445).

Variable	*β*	*T*	*p*
My usual daily routine	0.217	4.297	0.000
My future efficiency	0.208	4.016	0.000
The values of our society	0.117	2.486	0.013
The mood/atmosphere in the team	0.135	2.192	0.029

## Discussion

Due to its relevance for affected employees and their employers there are an ever-increasing number of studies investigating presenteeism. The majority of them have focused on antecedents as compared to consequences of the behavior (e.g., Miraglia and Johns, 2016; Lohaus and Habermann, 2019). With regard to previous research on consequences, it is obvious that it has usually been about the unfavorable health-related short-term and long-term effects for the individual or the economic impact for the organization (Lu and Cooper, 2022). Only recently, have (potential) positive effects of sickness presenteeism gained momentum (e.g., Karanika-Murray and Biron, 2020; Lohaus et al., 2021; Wang et al., 2022).

Within this context, the current study concentrated on two aspects that have received less attention so far. First, we simultaneously studied a variety of (potential) positive and negative effects of presenteeism, and second, we did that explicitly from the perspective of employees concerned. This subjective perspective is particularly important because in many cases of illness, individuals make a choice between presenteeism and absenteeism based on their assessment of the consequences. The goals of the study, to explain how perceptions of effects are related to illness behavior, were mostly met. In summary, the results of the study show that affected individuals opted for absenteeism in the case of heavier weighted perceived negative consequences, and presenteeism is more likely to be chosen in the case of more positively experienced or anticipated effects. It appears that only a relatively small number of specific effects are considered in the decision-making process. Interestingly, one’s own health does not play a decisive role. The results will be discussed in detail below along the structure of hypotheses and research questions.

### Perception of overall effects and their relation to behavior

Our presumption that the more positively participants perceive the effects of presenteeism, the higher is their presenteeism propensity (hypothesis 1) was confirmed. This result is consistent with the postulates of the HBM (Rosenstock, 1966; Rosenstock et al., 1988). The correlations between perceived benefits and behavior were equal and greater than those reported in the meta-analyses by Harrison et al. (1992) and Carpenter (2010). The difference could be due to the fact that the meta-analyses probably included benefits and health-related behaviors of a broader and less specific operationalization than we used in our study, which might result in lower correlations.

The second hypothesis stated that people who had enacted presenteeism assessed the presented effects of this behavior differently from those who had not worked when ill. Results supported the assumption derived from the HBM. The findings of the first two hypotheses are in accord with the suppositions of more general expectancy-value-models such as the expectancy theory of Vroom (1964, 1995). Cooper and Lu (2016) have already pointed out that expectations direct behavior and that people show presenteeism because they expect positive effects from it. This was confirmed in a VIE-based vignette study (Lohaus and Habermann, 2021), which showed that direction and strength of perceived effects significantly predicted the decision for presenteeism or absenteeism.

Hypothesis 3 covered the question whether affected employees act consistent with their perception of effects. Hypothesis 3a stated that people who reported presenteeism rated the overall effects of their behavior positively. This hypothesis was partly confirmed, i.e., when calculating the mean of the evaluations across all 45 effects. This finding fits those of the first and second hypothesis. However, the hypothesis was questioned with respect to the single-item measure of effects, which was slightly negative. The disparity between the two measures can probably be attributed to the fact that different aspects were taken into account. The single item expresses more strongly the attitude as a whole, which is not differentiated in terms of target or content and specificity (Judge and Kammeyer-Mueller, 2012), whereas the mean across all effects differentiates, but at the same time determines and limits the content to the effects that were included in the item pool. Thus, it might be assumed that participants who were asked for their overall assessment after having rated the 45 specific effects referred to them in their single-item rating. In contrast, those who rated the overall effect before being presented with the specific effects made an *ad hoc* decision and presumable took into account not only less than 45 effects but considered those who were more salient to them. The salience of specific aspects in arriving at the overall judgment may be influenced both by thoughts elicited by the instruction of the survey and by recent experience in one’s jobs, such as attendance/absence cultures (Xie and Johns, 2000; Ruhle and Süß, 2020) or task cohesion (Marques-Quinteiro et al., 2019).

Analogous to the first part of the hypothesis, we had expected that individuals who chose absenteeism would rate the effects of presenteeism negatively (Hypothesis 3b). Results confirmed the assumption for both measures of effects and hence, validate the findings of the first and second hypotheses. According to the HBM, it is assumed that individuals only opt for behavior that seems to provide sufficient benefit for them. Obviously, participants acted consistent with their beliefs in that they perceived potential effects of presenteeism as negative and consequently chose absenteeism in case of sickness.

### Relationship of specific effects with behavior and overall assessment of effects

With our first research question, we wanted to investigate which of the 45 specific effects of presenteeism were most important to explain the choice between presenteeism and absenteeism in case of sickness. Five effects were identified: one’s perception as reliable, the atmosphere in the team, the health of the team, the fulfillment of one’s job tasks, and one’s future performance capacity.

The inspection of the differences in mean evaluations across both subsamples seems to point at interesting disparities and rather consistent patterns. “Presenteeists” viewed all effects more positively than “absenteeists.” Further, there is a tendency to evaluate task-related effects (perception as reliable and completion of tasks) as clearly positive, while participants opting for absenteeism viewed them as rather negative or neutral. On the other hand, “absenteeists” seemed to care for their teammates’ health, while participants with a high degree of presenteeism perceived the effect near zero. One’s future efficiency suggests a less clear interpretation. The effect might have been understood as relating to one’s health-related performance capacity for those who never opted for presenteeism while people who engaged in presenteeism might have understood it as an indicator that refers to their ability, based on their presence, to complete their work tasks in a timely and appropriate manner. Participants with no presenteeism rated its influence on the mood in the team slightly negative whereas those with a high degree of presenteeism assessed it slightly positive. Employees deciding for absenteeism may have the idea that their presenteeism is stressing out colleagues because they are worried about catching the disease. They might also feel that presenteeism puts a strain on the atmosphere if the team members expect mistakes to be made because of reduced performance capacity due to illness. Further, it is conceivable that attendance despite illness is not appreciated by teammates because it contributes to a presenteeism culture. That is, the sick individual sets an example, which could lead to everyone feeling obliged to work when sick instead of taking a rest. On the other hand, individuals opting for presenteeism might want to avoid giving colleagues extra work due to the need to substitute for them.

We controlled for two directly work-related impact factors, namely work load and adjustment latitude. Both were related to presenteeism in earlier research (e.g., Gerich, 2014; Jourdain and Vézina, 2014; Baeriswyl et al., 2017; Saijo et al., 2017), yet not in our study. The non-correlation of these control variables in the current study (see test of Hypothesis 1) might be due to the more relevant impact of organization-related factors such as presence or absence cultures. These have previously been identified as important correlates of presenteeism (Miraglia and Johns, 2016). Attendance cultures (Xie and Johns, 2000; Ruhle and Süß, 2020) might shape the behavior in general but we did not control for this variable. We suggest considering this potential influence in future research.

With regard to person-related factors, we considered subjective health as a strong correlate of presenteeism (Miraglia and Johns, 2016). However, it was unrelated to presenteeism in the current study. The difference in findings can be explained by the different operationalizations of presenteeism. Miraglia and Johns used presenteeism days. For this measure, it is self-evident that it correlates with health because individuals who report more sick days may also show more presenteeism, while individuals with few sick days cannot show much presenteeism. We used presenteeism propensity, i.e., the proportion of presenteeism days out of all sick days. This measure is relative and therefore independent of the individual’s health status.

Surprisingly, the health of the affected employee, which was covered with several items (e.g., my physical health, my mental health, my stress, and my vulnerability) did not play a decisive role. This is astonishing because health was identified as the most important correlate of presenteeism (Miraglia and Johns, 2016). One explanatory approach could be to refer to original economic models that describe the valuation of consequences as a function of when they occur (Jungermann et al., 2010). Such models assume that each consequence has a timeless value, which is weighted by a subjective factor that represents the importance of when the consequence occurs. The prevailing discounting model assumes that the value of a consequence decreases as the time lag between the current event and its consequences increases. Discounting rates have been studied with respect to health-related decisions (Chapman, 2004). Urminsky and Zauberman (2015) point out that long-term health benefits are often perceived as more uncertain than present consequences of health-related behavior. Compared to other possible consequences, such as atmosphere in the team or completion of time-sensitive work tasks, for which effects are immediately experienced, health-related consequences may be perceived as less significant (in terms of importance and time of occurrence). Looking at the mean differences of the most relevant items (Table 4), it is noticeable that individuals who showed a high level of presenteeism rate two statements particularly highly that are directly related to the completion of their work tasks. In contrast, those who did not exhibit presenteeism rated the negative consequences for the health of others as particularly strong, as well as for their own efficiency. This finding could indicate that they feel a high responsibility for maintaining performance in the team.

Our second research question was twofold. In a first step, we wanted to find out to what degree the 44 specific effects of presenteeism (one was excluded from the analysis due to high intercorrelations with other effects) are able to explain the single-item overall assessment of effects. The analysis revealed a high amount of explained variance. We take this result as confirmation that our pool of items was successful in capturing the main potential consequences of presenteeism. In a second step, those specific effects contributing most to the overall assessment were identified. Four effects came to the fore (two of which were also found as explanatory variables with regard to research question 1): one’s usual daily routine, one’s future efficiency, the values of the society, and the atmosphere in the team. One’s future efficiency refers to the individual’s capacity to meet job demands in the long term. It is crucial for further advancement and social standing in the organization as well as the employability of the individual. It is therefore to be expected that the evaluation of one’s own future efficiency has a major influence on the decision for or against presenteeism. Individuals who believe that their efficiency will not be impaired by presenteeism and who feel capable to meet their job obligations despite health impairments will be more likely to choose presenteeism than individuals who perceive a higher risk that their future efficiency will be reduced if they do not take a rest. Two aspects (the impact on daily routine, the mood in the team) concern immediate consequences of presenteeism. Consistent with accounting models, this could indicate that care is taken to avoid immediate negative consequences when deciding for or against presenteeism. Less comprehensible in this context is the significance of the consequences for the values of society. This is a medium-term, if not long-term potential effect. It might be assumed that those affected take particular account of personal standards (e.g., feeling guilty for not working, Brosi and Gerpott, 2022), which they perceive to be in line with society’s values when making their decision. The relationship of this item with the single-item overall assessment may also indicate that attendance behavior depends on the individual’s general attitude toward it (apart from such severe illnesses or contagious diseases where the individual has no capacity to meet job demands).

### Theoretical implications

The results of the current study confirm the applicability of the HBM and the VIE to the explanation of sickness presenteeism. Meta-analyses on the HBM show a medium association of perceived benefits and health-related behaviors (Harrison et al., 1992; Carpenter, 2010). The current study also confirmed such a relationship, which was somewhat more pronounced (*r* ≥ 0.30). Controlling for important factors that may influence the perception of the effects (i.e., workload, job control, and subjective health) resulted in an even slightly higher correlation. That means workload, job control, and subjective health were not pivotal for the individuals’ decision-making process. The findings support the notion that employees take into consideration a variety of perceived consequences when deciding for or against working despite sickness.

Individuals who consistently choose absenteeism unanimously rate the consequences of presenteeism negatively. The picture is less clear for individuals who exhibit presenteeism. They see the effects as only partially positive. This finding indicates that in the future it should be investigated on the basis of the individual case of illness in each case, which consequences were expected at that time and how they are related to the behavior in the specific case. Enriching for the understanding of presenteeism is the result that individuals with a high level of presenteeism apparently give more weight to the positive consequences of the completion of their work tasks than to possible negative consequences for their health. This finding is consistent with the health-performance framework (Karanika-Murray and Biron, 2020), which posits a simultaneous consideration of the need for rest and the performance requirements.

### Practical implications

Practical implications relate to the awareness of the effects of sickness presenteeism on the side of the employee, to the management of presenteeism in organizations respectively, and to physicians’ understanding of the phenomenon. Individuals showing presenteeism may not always be aware of the bundle of potential consequences behind their decision. Notwithstanding the subjective perception of effects when showing up for work in case of illness, employees might profit from insight into the general driving forces that generate the decision and find reason to review their situation. In dealing with presenteeism, the management might profit from knowing what expected consequences trigger the decision for working despite illness. As the major effects revealed in our study do not expressively relate to the own health of the employee, superiors should be aware of the at least long-term health consequences of the behavior and should not be satisfied with immediate economic advantages for the organization. Therefore, the common approach of asking employees who feel ill to call in sick does not seem advisable. Instead, managers should be trained to deal with contradictory consequences. This training should not be limited to recognizing warning signs of presenteeism (CIPD, 2022), but should enable a differentiated approach to the conditions that appear to be associated with presenteeism and the measures to be taken (Godoy, 2016). This includes, for example, making it clear to employees that absence due to illness has no influence on their assessment as reliable and committed. Short-term productivity benefits of presenteeism-showing employees over total productivity loss in case of their absenteeism might prove no sufficient compensation for long-term shortages of experienced personnel due to subsequent serious illness. Thus, it does not seem advisable to use blanket measures such as attendance bonuses (e.g., dpa/lhe, 04/22/2022), as in individual cases, these could lead to employees substituting necessary absence to cure their illness with attendance (Aronsson et al., 2021). For physicians who are primarily concerned with the somatic of disease, the results of our study may prompt them to consider benefits of presenteeism as perceived by their patients and tailor their treatment accordingly. Knowledge of the factors that might cause patients not to use or to only partially use recommended remedies could enable physicians to discuss benefits and costs of presenteeism and absenteeism on a more comprehensive basis.

### Limitations, strengths, and future directions

We used a cross-sectional research design, so no causal conclusions can be drawn. Participants, the only source of data, rated the illness-related questions and those on the effects of their behavior in retrospect, which may incorporate recall errors. The study is based on subjective assessments of the respondents. However, this possible limitation was deliberately accepted, since the study’s research question focused on expectations that influence the respondents’ behavior. The study was conducted in Germany, whose specific legislation with regard to continued payment of wages in the event of illness means that employees feel less threatened in their existence by (short-term) absence in case of illness than in other countries. Capturing the expected financial effects of presenteeism could lead to different results for employees in other countries. We collected the data *via* an online panel provider which gave us access to a broad pool of employees (Aguinis and Bradley, 2014; Landers and Behrend, 2015). This procedure has a favorable impact on the representativeness and generalizability of the results (Cheung et al., 2017). Consistent with recommendations to ensure data quality in online studies, we both incorporated items to control for attention and excluded subjects from the analysis who had unrealistically short completion times (Cheung et al., 2017; Porter et al., 2019). In this study, we referred to the gray area of sickness between the poles of complete health and severe health impairments that require professional medical treatment. This gray area includes health restrictions that provide affected employees with a justification, but not a compelling reason, for taking sick leave. Thus, it does not refer to the state of certified inability to work (Gemeinsamer Bundesausschuss, 2022). However, we did not collect data on the type of illness, i.e., acute or chronic, physical or mental illness, which might have an impact on sickness behavior. Since the decision to work or not in case of illness depends on the individual’s evaluation of their domain-specific capacities to meet the job demands (Linden et al., 2010), the type of illness and its resulting limitations in capacity should be considered in future research.

Methodological strengths of the study lie in the systematic development of the stimulus material and the simultaneous investigation of positive and negative effects of presenteeism with a focus on subjective perceptions. Our focusing on the data analysis to sick employees provides relevant information for the personnel management and the company health care system as to how much support employees need with regard to their health without neglecting their professional goals and what kind of support this could be (Kooij et al., 2014).

Concerning future research, it seems useful to investigate the phenomenon of presenteeism with regard to its expression of a general work attitude, as has already been proposed (Krane et al., 2014; Rebmann et al., 2016; Lohaus and Habermann, 2019). One indicator that points in this direction is the finding of the current study that participants who never opted for presenteeism rated 42 out of 45 specific effects negatively. Only three effects were descriptively positive. These were one’s income, one’s reputation with their manager, and the social security systems. Some of our findings point to the possibility that the evaluation of effects, and the attendance behavior is shaped by the employers’ culture (Xie and Johns, 2000; Ruhle and Süß, 2020). Thus, we recommend that future research should focus on absence/presence cultures as a potential mediating variable. Further, to gain a more detailed picture of the impact at the individual level, it would be useful to ask individuals to refer to the most recent instance of illness to assess the consequences of their behavior at that time. It should be noted, however, that the latter approach is then less suitable for capturing long-term consequences.

## Data availability statement

The raw data supporting the conclusions of this article will be made available by the authors, without undue reservation.

## Ethics statement

Ethical review and approval was not required for the study on human participants in accordance with the local legislation and institutional requirements. The patients/participants provided their written informed consent to participate in this study.

## Author contributions

DL and WH contributed to the study conception and design, development of the stimulus material, data collection, and analysis, and wrote the manuscript. MN assisted in the technical design of the survey and the handling with the external service provider, and advised on the data analysis and its processing in SPSS. All authors contributed to the article and approved the submitted version.

## Funding

We acknowledge support by the Open Access Publishing Fund of Hochschule Darmstadt—University of Applied Sciences.

## Conflict of interest

WH was employed by H&L Karriereberatung.

The remaining authors declare that the research was conducted in the absence of any commercial or financial relationships that could be construed as a potential conflict of interest.

## Publisher’s note

All claims expressed in this article are solely those of the authors and do not necessarily represent those of their affiliated organizations, or those of the publisher, the editors and the reviewers. Any product that may be evaluated in this article, or claim that may be made by its manufacturer, is not guaranteed or endorsed by the publisher.
